# Fact or Fiction? Examining the Veracity of Common Myths Related to 7-meter Throws in Handball

**DOI:** 10.5114/jhk/204312

**Published:** 2025-09-23

**Authors:** Aron Laxdal, Per Thomas Byrkjedal, Andreas Ivarsson, Olafur Sigurgeirsson, Tommy Haugen

**Affiliations:** 1 Department of Sport Science and Physical Education, University of Agder, Kristiansand, Norway.; 2 Center of Research on Welfare, Health and Sport, Halmstad University, Halmstad, Sweden.; 3 HBStatz, Seltjarnarnes, Iceland.

**Keywords:** confirmation bias, hot hand fallacy, momentum, psychological safety, self-taking

## Abstract

There are several long-standing beliefs that persist in sport despite little or no evidence of them being true. The aim of this study was to assess the veracity of two such beliefs related to 7-meter (7-m) throws in handball: 1) that the fouled players should not take their own 7-m shots as their ability to score is compromised, and 2) that changing the 7-m shooter after a miss is more likely to lead to a goal than letting the player who missed try again. To assess the veracity of these beliefs 10,593 7-m shots from the Icelandic elite division were analyzed using a Bayesian three-level analysis. The results revealed that neither taking your own 7-m shot after being fouled nor taking a 7-m shot after missing the attempt appeared to have a negative effect on the outcome of 7-m shots. Coaches should therefore be able to let their most proficient 7-m shooters take any 7-m shot, regardless of preceding events, without any functional cost.

## Introduction

There are several long-standing beliefs that persist in sport despite little or no evidence of them being true. They are kept alive by heuristics and confirmation biases and passed on from one generation to the next. Some beliefs even persist after being refuted by research, as the research is often inaccessible, and hard to comprehend (Buchheit, 2017; Halperin, 2018; Keegan et al., 2018). Consequently, practitioners tend to add more weight to their own assumptions and lived experiences as findings from scientific research are often perceived as sanitized, unrealistic, and irrelevant (Buchheit, 2017; Fullagar et al., 2019; Keegan et al., 2018). Examples of such beliefs are icing free-throw shooters in basketball (i.e., when the opposing team takes a time-out before a player performs a free-throw in an attempt to undermine performance), and clutchness (i.e., superior performances during high pressure situations, generally during the latter stages of games), which are generally accepted as real phenomena, even though little or no clear empirical basis for the claims have been found (note that icing has been found to be effective when it comes to field-goal kickers in American football [Goldschmied et al., 2010, 2023; Schweickle et al., 2021]).

In handball two such beliefs are frequently applied to 7-m shots (i.e., the penalty shot in handball, awarded to the attacking team when a player is robbed of a clear goalscoring opportunity). The first one concerns players taking their own 7-m shots after being fouled. The belief centres around the claim that the short-term spike in activation that follows being fouled will supposedly reduce the player’s ability to score from a shot that requires increased focus and attention. However, previous studies on soccer have found no indications of this belief being true (Kuss et al., 2007; Lackner and Sonnabend, 2021).

The outcome of free-throws in basketball, which are similar to 7-m throws in handball, have been found to be impacted by acute physiological stress (Padulo et al., 2015; Schmitzhaus et al., 2022; Uygur et al., 2010). However, an important distinction has to be made between basketball and handball, as it is compulsory for basketball players to take their own free-throws. Contrastingly, 7-m shooters in handball are usually specialists at that shot type, who are chosen by the coach to execute that specific task. They have usually proven their proficiency over a prolonged period and shown that they can withstand the pressure associated with the situation. Additionally, since acute distress was not found to affect shooting mechanics in basketball (Uygur et al., 2010), there is reason to believe that the same may apply in handball.

The second belief concerns changing the 7-m shooter after an unsuccessful attempt. Most handball teams have one or more designated 7-m specialists that train the penalty shot specifically and have usually handled that responsibility for some time. None of those players score from all their 7-m shots. In fact, the average success rate of 7-m shots is around 75% (Debanne et al., 2018; Laxdal et al., 2022a; Lobinger et al., 2014). Changing from the team’s most proficient 7-m shooter to the team’s second most proficient 7-m shooter because of a single miss can therefore appear counterproductive. Especially considering the fact that the best players in other sports have been found to be more likely to score after missing a shot (Chang, 2019).

In line with the hot hand theory and psychological momentum this belief centres around the assumption that success breeds success and that failure breeds failure (Descamps et al., 2022; Iso-Ahola and Dotson, 2014; Zhao and Zhang, 2023), the underlying tenet being that subsequent performances are somehow dependent on the previous performance. Initial success or failure would therefore either result in an increase or a decrease in confidence, concentration, and expectation of success (Iso-Ahola and Dotson, 2014). However, constantly changing 7-m shooters after misses could also add undue pressure to a situation that is already perceived as stressful (Goldschmied et al., 2021), possibly affecting the shooters’ ability to put the ball past the keeper. In such moments, a psychologically safe performance environment and support from coaches and teammates would probably be preferable (Buñuel et al., 2021; Gosai et al., 2021; Vella et al., 2022). The idea being that an environment where failure is viewed as a natural part of performing, where the 7-m shooters feel that they are supported irrespective of the outcome of any given shot, would decrease the stress and anxiety associated with the task (Vella et al., 2022).

The aim of this study was to assess the veracity of the aforementioned beliefs. We hypothesized that neither claim would withstand scrutiny and that 7-m shooters who either took their own 7-m shots after being fouled or took 7-m shots after having missed their previous attempt in the same game, would not perform worse than their replacement shooters.

## Methods

### Data and Procedures

All 7-m shots in the semi-professional elite handball league in Iceland from 2018 to 2024 were registered into the HBStatz (www.hbstatz.is) system in situ by representatives from the home teams. These representatives were familiar with the platform and had received training prior to the data collection. A total of 10,593 7-m shots were registered, 6,366 in the male league and 4,227 in the female league. In an effort to increase the reliability of the collected data, HBStatz utilizes various data quality processes. All HBStatz reports are cross-referenced with the official game reports and logical tests are performed on all games to ensure that the reports balance out (e.g., the number of 7-m throws any given team receives should match the number of goals from 7-m throws combined with the number of missed 7-m throws). Any games that are flagged during these data quality processes are reviewed, and recompiled if needed. Additional quality controls are done at random in real time by representatives from HBStatz. Þorgeirsson et al. (2022) tested the reliability of the data delivered by HBStatz, finding the data to be reliable across a multitude of variables. HBStatz is the official statistics partner of the Icelandic handball association and all the data that were used in this study are openly available online and part of the public domain. No ethical approval was therefore needed, and informed consent was not obtained from the players.

**Figure 1:**
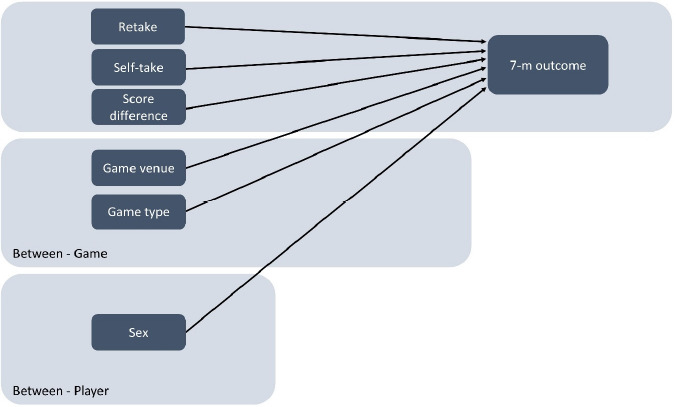
A Directed Acyclic Graph illustrating the hypothesised causal associations between the variables.

The main variables of interest in this study were the outcome of the 7-m shots (measured dichotomously as a goal or a miss), whether the shooter was awarded the 7-m shot him- or herself, and whether a previous miss within the same game resulted in a change in 7-m shooters or not (i.e., did the coach choose to change 7-m shooters if the previous 7-m shot was unsuccessful?). Other variables of interest were the sex of the shooter (whether the shooter played in the male or the female league), game venue (whether the game was played at home or away), game type (whether the game was played during the regular season or the playoffs), and score differential (the number of goals the 7-m shooter’s team was leading or trailing by when the 7-m shot was executed). These variables were included as covariates to account for possible differences between the sexes, and the varying criticality of different games and periods within games (e.g., Oliveira et al., 2012). The proposed causal structure of the associations is illustrated in Figure 1.

### Statistical Analysis

**Figure 2:**
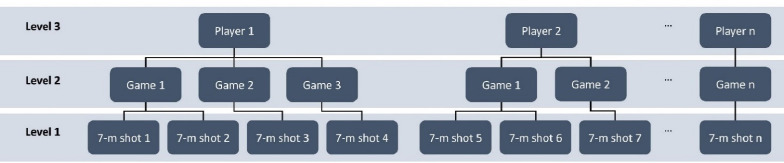
An illustration delineating the different levels in the multilevel setup.

Descriptive frequencies and contingency tables were calculated using SPSS (version 28.0; IBM Corp). Statistical significance was accepted at *p* < 0.05 and the benchmarks for phi (Φ) were 0.00–0.10 = negligible, 0.10–0.20 = weak, 0.20–0.40 = moderate, 0.40–0.60 = relatively strong, 0.60–0.80 = strong, and 0.80–1.00 = very strong (Lee, 2016). To analyse the relationship between self-taking, changing 7-m shooters after misses and the outcome of 7-m shots, a Bayesian three-level analysis was performed in Mplus (version 8.4; Muthén and Muthén, 2017). Within the model, the outcome of the 7-m shots (level one) were nested within games (level two). Games were in turn nested in players (level three). Self-taking and retaking after a miss were, together with the score differential, included as covariates on level one in the model. The game venue and the game type were controlled for on level two and sex was controlled for on level three. For an illustration of the multilevel model see Figure 2.

A Bayesian estimator, using the default median of the posterior distribution, was used for the analysis (for more information on the difference between the Bayesian estimator and the more traditional frequentist estimator see Wagenmakers et al., 2018). The WAMBS-checklist was therefore used to guide the report of the analytical strategy and the results. We ran the analysis with four Markov chains for each of the specified parameters. Chain convergence was assessed by applying the Gelman and Rubin convergence diagnostic (this process is described in the Mplus manual). However, we applied a stricter convergence criterion than the default setting (0.01 instead of 0.05). The Gelman and Rubin diagnostic indicated adequate convergence for each of the four chains. In the next step, we visually inspected the trace plots for each parameter. In this inspection, all four chains showed adequate convergence for each of the parameters. To validate convergence and rule out potential errors related to local convergence we ran the analysis again using 200,000 interactions. The Gelman and Rubin convergence diagnosis indicated convergence for all parameters in the analysis. Because of the novel design we decided to rely on the default prior settings (N [0, 10^10^]) of the software for the variance in all parameters. To assess the model fit, the posterior predictive *p* (PP *p*) value and its accompanying 95% confidence interval were used (Muthén and Asparouhov, 2012). Credibility Intervals (CI) were obtained for all parameters within the model. A 95% CI not containing zero was considered to indicate a statistically significant result (Zyphur and Oswald, 2015). For each of the regression parameters Beta values (β) were obtained.

## Results

Out of the 10,593 7-m shots, 2,591 (25%) were taken by the fouled player (i.e., self-taking). A total of 2,587 of the 7-m shots resulted in a miss, with 1,929 of them being followed by a subsequent 7-m shot in the same game, giving the coach the opportunity to either choose a new 7-m shooter or allowing the previous shooter to try again (i.e., the opportunity to retake). A majority of those misses resulted in a change of the shooter (1,234 vs. 695). The scoring efficiency from self-taking and changing shooters was the same (76%; *χ*^2^ [1, N = 10,593] = 0.26, *p* = 0.61), while the scoring efficiency of retaking after a miss was 78%, and 74% with a different shooter. The difference between retaking and changing shooters was found to be statistically significant (*χ*^2^ [1, N = 1,929] = 3.95, *p* = 0.047), but the effect size was negligible (Φ = 0.045, *p* = 0.047).

The three-level model showed a good fit to the data (PP *p* = 0.52, 95% CI = [−13.08, 13.78]), and there were no effects of self-taking (level one; β = 0.04, 95% CI = [−0.01, 0.08]), retaking after a miss (level one; 0.04, 95% CI = [−0.01, 0.09]) or score differential (level one; 0.04, 95% CI = [−0.01, 0.09]) on the outcome of 7-m shots. In the between matches comparison (level two), no effects of the game venue (β = −0.02, 95% CI = [−0.29, 0.22]) or the game type (β = −0.02, 95% CI = [−0.25, 0.30]) were found on the outcome of 7-m shots. There was no association between sex and the success rate (β = −0.06, 95% CI = [−0.49, 0.40]).

## Discussion

This study assessed the veracity of two long-standing beliefs related to 7-m throws in handball, specifically, that 7-m shooters should avoid both self-taking 7-m shots and re-taking 7-m shots after a miss. No evidence was found for either belief in the current analysis, indicating that the outcome of 7-m throws is, to a larger extent, determined by other factors. These results are therefore aligned with many other efforts to test the veracity of long-held beliefs in sport (Casey et al., 2023; Goldschmied et al., 2023; Kuss et al., 2007; Schwickle et al., 2021).

These results are in line with previous studies on self-taking, where taking your own penalties after being fouled was not found to have an association with the outcome in German soccer (Kuss et al., 2007; Lackner and Sonnabend, 2021). In further alignment with Kuss et al. (2007), no covariates external to the shooters were found to have any bearing on the outcome. In other words, internal factors such as the shooters technical abilities and shooting repertoire appear to be the decisive factors. These results are therefore also in alignment with the findings of Krumer (2020), who found that the outcome of penalties in soccer was to a large degree determined by the shooters’ level of proficiency.

While comparing unhindered free shots (i.e., penalties, free-throws, 7-m shots) across sports is meaningful, it is important to note the various contextual dissimilarities that distinguish between each shot type. Handball is more similar to basketball than soccer when it comes to the frequency of penalty shots (i.e., 7-m throws and free-throws). While free-throws are quite frequent in basketball and handball (Kozar et al., 1994; Laxdal et al., 2022a, 2022b), they are an infrequent part of gameplay in soccer and occur approximately once every four games (Dalton et al., 2015). Basketball is nevertheless dissimilar to handball when it comes to the complexity of the throw and the fact that basketball players are required to take their own free-throws. While 7-m shots are interactive and complex, meaning that success is not only dependent on the shooters’ execution of the shot, free-throws are non-interactive and constrained, meaning that a perfectly executed shot will end up in the basket.

Having the choice to select yourself in or out of a task that you are either comfortable or uncomfortable with executing will likely affect your association with that task. While 7-m shooters will practice with a performance-approach, many basketball players will practice free-throws with an avoidance-approach (Elliot, 2006). In other words, the 7-m shooters train towards excellence, while many of the free-throw shooters train to avoid failure. This difference is captured well by Maher et al. (2020) who studied how basketball players coped with free-throw-related pressure and anxiety. It is apparent that some of the players in that study would gladly opt out of their free-throw responsibilities if they could, as the act was perceived as stress-inducing, and they appeared to be afraid of failure. In contrast, handball players who do not have the mental fortitude to take 7-m shots, will not be selected. They do not have to deal with being targeted by the opposing team either, i.e., the hack-a-Shaq strategy (Gómez et al., 2018). Interestingly, the success rate in all three sports is around 70–80% (Brinkschulte et al., 2020; Gómez et al., 2018; Laxdal et al., 2022a).

The literature on psychological momentum in sports is indecisive to say the least, with varying conclusions based on different philosophical and statistical standpoints (Bar-Eli et al., 2006; Miller and Sanjurjo, 2021; Morgulev et al., 2020; Ritzwoller and Romano, 2022). While some claim that psychological momentum exists (Descamps et al., 2022; Miller and Sanjurjo, 2021; Ram et al., 2022; Zhao and Zhang, 2023), others claim that variation and periods of better and worse performances are natural in sports (Chang, 2019; Csapo et al., 2015; Morgulev et al., 2020; Ritzwoller and Romano, 2022; Steeger et al., 2021). Regardless of this larger debate, the results of this study indicate that missing a 7-m shot does not affect the outcome of the subsequent attempt. Some might even have expected retaking to have a positive effect on the outcome of the shot, especially in light of the positive corollaries associated with psychological safety. However, Bühren and Gabriel (2023) found that 7-m shooters thrived under pressure, meaning that the added burden associated with losing the 7-m duties might not be debilitating. Bühren and Gabriel’s (2023) findings backed up the results of Debanne et al. (2018), who found that a high fit between situational focus and reward structure led to better 7-m outcomes in the French male handball league. Findings from basketball, where free-throw efficiency has been found to go down during critical moments late in games (Gómez et al., 2018), may therefore not be relevant here. The findings of both Bühren and Gabriel (2023), and Debanne et al. (2018) run counter to the findings of Jordet and Hartmann (2008), who claim that pressure has a negative effect on goalscoring from penalties in penalty shootouts in soccer. Nevertheless, penalties in penalty shootouts in soccer should be considered substantially different from in-game 7-m shots in handball. Additionally, 7-m shooters are much more accustomed to the task than most penalty kickers are.

The findings of this study also run somewhat counter to the findings of Green and Zwiebel (2018), who found significant hot hand effects in baseball during the period from 2000 to 2011. Their explanation for the large effect was related to reduced ability of the opponents to make tactical changes that could counteract the hot hand. However, if that was true, the same trend should be apparent in this sample. In fact, the dynamic between the 7-m shooter and the goalkeeper bares striking structural resemblance to the dynamic between the pitcher and the batter. Then again, Glazer and Goldberg (2020) found no evidence of a hot batter effect in the same sport during the 2018 season.

## Limitations and Future Research

Even though the studied data are large and robust, the number of datapoints concerning 7-m shooter changes is relatively small. Nevertheless, the sample is within the recommendations for nested data (Arend and Schäfer, 2019). Seeing as the data were collected by individuals unrelated to the study, the authors had relatively limited control over the data collection process. However, the strict data quality mechanism in place at HBStatz increases the reliability of the data. Additional covariates that could have affected the outcome of the 7-m shots (e.g., feints, ball speed, time from the referee’s whistle to shot execution) were not collected and could therefore not be included in the analysis. Furthermore, Ritzwoller and Romano (2022) argued that momentum affected players differently, and that analysing shots as repeated random independent trials could miss evidence of streaks. This concern was addressed through the nested structure of the analysis. Covariates that some might argue could affect or mask the findings of the study, such as the shooters’ playing position, body height, body mass and the dominant throwing arm, were not included as they have been found to be inconsequential when it comes to 7-m shot efficiency (Laxdal and Haugen, 2024).

This study utilized data from leagues that were ranked 25^th^ (female league) and 27^th^ (male league) on the European Handball Federation’s coefficient (European Handball Federation, 2023). Even though previous research has not found differences in cognitive traits between elite and sub-elite players (Blecharz et al., 2022), future research should address whether these findings apply at other performance levels. Interestingly, previous studies have shown that 7-m shot efficiency is relatively stable across performance levels and contexts (see e.g., Bühren and Gabriel, 2023 [77%]; Debanne et al., 2018 [77%]; Laxdal and Haugen, 2024 [78%]; i.e., similar to this study). Qualitative interviews with 7-m shooters on their thought processes, strategies and coping mechanisms related to taking 7-m shots would add valuable nuance to the discussion.

## Conclusions

Neither of the studied beliefs appear to be true at the semi-professional level and 7-m shooters appear to be able to withstand the pressure of the task at hand irrespective of preceding events. Handball coaches should therefore be able to let their most proficient 7-m shooters take any 7-m shot, with no regard for preceding events, without any functional cost. However, the relative difference in quality between the first-choice shooter and his or her replacement on each team will vary and should be considered when deciding whether to retain or change shooters. These results may not be transferrable to sports where the fouled players are required to take the subsequent shot themselves, as these specialized shooters have been selected for the task based on their ability and previous record.

## Data Availability

Data is openly available at https://doi.org/10.6084/m9.figshare.21026806.v3
